# FOXD3-induced miR-133a blocks progression and metastasis of colorectal cancer through regulating UBA2

**DOI:** 10.7150/jca.60647

**Published:** 2021-08-25

**Authors:** Yuanfang Cheng, Yajuan Wang, Yuanzun Cheng, Quanzhong Yang, Lejing Zhang, Zhaoxi Li, Jiancheng Cheng

**Affiliations:** 1 Sanquan College of Xinxiang Medical University, west of Changjiang Avenue, Pingyuan New Area, Xinxiang City, Henan Province, China.; 2 College of Nursing, LuoYang Polytechic, Luoyang, 471000, Henan, China.; 3 Department of Cardiovascular Surgery, Zhengzhou Central Hospital Affiliated to Zhengzhou University, Zhengzhou 450000, China.

**Keywords:** MiR-133a, UBA2, Colorectal Cancer, FOXD3, GEO, ArrayExpress

## Abstract

**Background and Aim:** Some studies have verified that miR-133a played an inhibitory role in several cancers. Whereas, the effect of miRNA-133a in colorectal cancer (CRC) has not been fully elucidated. Our study aims to confirm UBA2 as a direct target gene of miRNA-133a and explore the upstream modulatory molecules of miR-133a. In addition, their impacts on the biological characteristics of CRC cells were assessed.

**Methods:** QRT-PCR analyzed miR-133a expression levels in colorectal cells including HCT116, SW48 cells and human normal colorectal cell line NCM460. A serial biological experiment assessed miR-133a effects on cell proliferation, migration, invasion and apoptosis capacities in HCT116 and SW48 cells. MiRNA targeting gene prediction and a dual luciferase assay were employed to confirm miR-133a-targeted UBA2. Transcription factors (TFs) FOXD3 was identified as an upstream regulator of miR-133a via JASPAR. The influence of miR-133a and FOXD3 on UBA2 expression was analyzed by qRT-PCR or western blot.

**Results:** miR-133a was lowly expressed in CRC cells. High miRNA-133a expression suppressed the proliferation, migration, invasion and enhanced apoptosis capacities of CRC cells. MiR-133a targeted the UBA2 mRNA 3ʹUTR area and reduced UBA2 protein expression. We also unveiled that FOXD3 high-expression significantly raised miR-133a expression and diminished UBA2 expression. We also discovered that high miR-133a expression augmented the effects of elevated FOXD3 expression on CRC cell proliferation, migration and invasion, whereas, low miR-133a expression generated the opposite outcomes.

**Conclusion:** FOXD3 induced miRNA-133a directly targeting UBA2 could affect the progression and growth of CRC.

## Introduction

CRC is the third most commonly diagnosed carcinoma and cause of cancer-related death in the United States 1. In spite of major advances in treatment containing surgery, chemotherapy and radiation treatment, the prognosis of CRC patients remains unfavorable 1. It is urgently needed to explore the molecular mechanisms of CRC growth and progression, which may be underlying therapeutic targets for CRC therapy.

MiRNAs are small (20-22 nucleotides), single stranded, non-coding RNA molecules that play critical roles in various biological processes through post-transcriptionally regulating gene expression 2. Evidence has demonstrated that miRNAs were tightly involved in the growth and development of tumors 3-5. Huang et al. revealed a dissecting miRNA signature in colorectal cancer progression and metastasis 6. Song et al. identified that FOXO3a-driven miRNA signatures suppressed VEGF-A/NRP1 signaling and breast cancer metastasis 7. Niu et al. revealed biological implications and clinical potential of metastasis-related miRNA in CRC 8. Meanwhile, downregulation of miR-133a has been reported in several carcinomas, such as oral squamous cell carcinoma 9, gastric cancer 10, esophageal squamous cell carcinoma 11. Besides, several reports discovered that TFs regulating miRNAs expression were strongly involved in carcinoma 12, 13. Therefore, the deregulation of TFs-miRNAs may serve as a crucial factor of aberrant miRNA expression in tumor. We disclosed that miR-133a was obviously downregulated in CRC across bioinformatics methods. However, the therapeutic impact and mechanisms of TFs-miRNAs in the progression of CRC required to be further explored.

Herein we unveiled that miR-133a was significantly low-expressed in CRC tissue samples and cells. We determined UBA2 as a direct target of miR-133a, and the expression of UBA2 was decreased by miR-133a overexpression. Both knockdown of miR-133a and overexpression of UBA2 in CRC cell lines promoted cell proliferation, invasion and migration and inhibited apoptosis. In addition, to investigate the upstream regulatory association of miR-133a, TFs that modulated promoters of miR-133a was discovered through JASPAR. Taken together, our study indicated that miR-133a induced by TFs could regulate the growth and development of CRC by reducing UBA2.

## Methods and Materials

### Bioinformatics data analyzing

Firstly, The GEO and ArrayExpress databases were searched to obtain raw miRNAs and clinical data for each CRC sample. Afterward, the normalization of the raw miRNA data was executed across the Robust Multi-Array Average (RMA) and Linear Models for Microarray (LIMMA) algorithm 14. Subsequently, Limma package 15 was adopted to unveil the differentially expressed miRNAs (DEMs) between the CRC tissues and normal tissues. P<0.05 and logC>1.3 exhibited a statistical significance. Then, we performed the overlapped analysis for CRC DEMs via E-MTAB-4036 in ArrayExpress database and GSE98406 in GEO database. Furthermore, the overlapped miRNAs were measured by adopting Venn diagram by vennDiagram package. Further, the volcano map was performed by utilizing ggplot2 package to test the DEMs' and differentially expressed genes' (DEGs) expression between the CRC tissues and the non-CRC tissues.

### Determination of miR-133a target genes

The candidate target genes of miR-133a were determined via four bioinformatics software including miRWalk2.0 16, TargetScan6.2 17, miRanda 18 and RNA22 19. Furthermore, the predicted and validated target genes were adopted to intersect with the DEGs in GSE156355 and GSE50117 sets from GEO database. Likewise, the DEGs between the CRC tissues and the non-CRC controls were uncovered on the basis of the above screening strategy 14. At last, the uncovered targeted genes were assessed across PCR and western blot assays.

### Cell Culture and Transfection

Human normal colorectal cell line NCM460 and CRC cell lines HCT116, SW48 were purchased from the American Type Culture Collection. All cells were cultured in DMEM medium (Gibco, USA) supplemented with 10% FBS (Gibco, USA) at 37 °C in a cell culture incubator containing 5% CO2.

The miR-133a mimic, the mimics negative control (miR-NC) and miR-133a inhibitor were purchased from GenePharma (Shanghai, China). The pcDNA (Vector) and pcDNA-FOXD3 overexpression (FOXD3-OE) plasmids were purchased by GenePharma (Shanghai, China). The pcDNA (Vector) and pcDNA-UBA2 overexpression (UBA2-OE) plasmids were synthesized by GenePharma (Shanghai, China). All transfections were carried out by using Lipofectamine 3000 reagent (Invitrogen) following the manufacturer's protocol. Post transfection 48 hours, cells were collected for testing transfection efficiency via QRT-PCR analysis.

### Western Blot

Total proteins were extracted via RIPA lysis buffer (Sangon Biotech) following the manufacturer's instruction. Then, the proteins were separated by applying 8-10% SDS-PAGE gel (Beyotime, China). Following the separation process, proteins were transferred to PVDF membranes. After blocking with 5% nonfat milk for 1 hour, the membrane was incubated overnight at 4 °C with primary antibody. Signals were visualized applying chemiluminescence (Tanon, China). Primary antibodies employed for immunoblotting were exhibited as followings: (GAPDH, 1:5000, ab8245, Abcam; UBA2, 1:2000, ab185955, Abcam; FOXD3, 1:800, ab64807, Abcam).

### Quantitative reverse transcription-polymerase chain reaction (QRT-PCR)

Total RNA extraction was conducted using TRIzol (Invitrogen, USA), according to the manufacturer's protocol. A reverse transcription kit (Invitrogen, USA) was used for reverse transcription in accordance with the manufacturer's instructions. Real-time PCR assay was carried out by employing the SYBR Premix Ex Taq II (TAKARA) on the basis of manufacturer's protocols. U6 and GAPDH were employed as the internal controls. Relative expression levels were measured applying the 2^‒ΔΔCt^ method. Primer sequences are placed in **Table 1**.

### Luciferase reporter assay

Starbase databasewas was applied to predict the underlying targeting interplays between UBA2 and miR-133a. To identify the association between miR-133a and UBA2, luciferase reporter assay was carried out. HCT116, SW48 cells were transfected using PMIR-REPORT luciferase vector (GenePharma, Shanghai, China) with wild-type (WT)-UBA2-3'UTR or mutant (MUT)-UBA2-3'UTR, miR-133a mimic or miR-133a inhibitor (miR-133a-KD) or miR-NC. Post transfection for 48h, luciferase activities were measured across the Dual-Luciferase Reporter Assay System in accordance to manufacturer's instructions.

### Transwell assay

Transwell assay were undertaken to test the migratory and invasive abilities of CRC cells. Cells were placed in the upper layer of the transwell chamber. Then, the lower chamber was filled with DMEM supplemented with 30% FBS. Post culture for 24 hours, cells on the upper parts were wiped out, while the invaded cells in the lower compartments were fixed and stained using 0.1% crystal violet, and then counted according to a high-power microscope.

### Cell proliferation assay

The proliferative capacity of CRC cells was assessed by 3‐(4, 5‐dimethylthiazol‐2‐yl)‐2, 5‐diphenyltetrazolium bromide (MTT) assay in accordance to the manufacturer's instructions. Post transfection for 48 h, the cells were added to 96‐well plates and placed into an incubator at 37 °C with 5% CO2. At the indicated time point (24 hours, 48 hours, 72 hours, 96 hours and 120 hours), cells were harvested and interacted with 10 ul of MTT (5 mg/mL). Following 4 hours' incubation at 37 °C, 100 ul DMSO (per well) was used to dissolve the formosan. At last, a microplate reader was employed to detect the absorbance values at 490 nm.

### Chromatin immunoprecipitation (ChIP) assay

ChIP analysis of miR-133a was undertaken via a kit (Beyotime, Shanghai, China) in accordance to manufacturer's instructions. Briefly, FOXD3 antibody was used for immunoprecipitation of chromatin, and IgG was negative control and anti-RNA Polymerase II was utilized as positive control. Analysis of isolated DNA through RT-qPCR by applying gene-specific primers.

### Statistical analysis

All statistical analyses were carried using GraphPad Prism 7.0. Data were exhibited as mean ± SEM of at least 3 times of independent experiments and statistical significance of the differences was calculated with the one-way analysis of variance or Student t-test. P value < 0.05 was considered to be statistically significant.

## Results

### MiR-133a expression was decreased and its high-expression was involved in favorable OS in CRC

**Figure 1:**
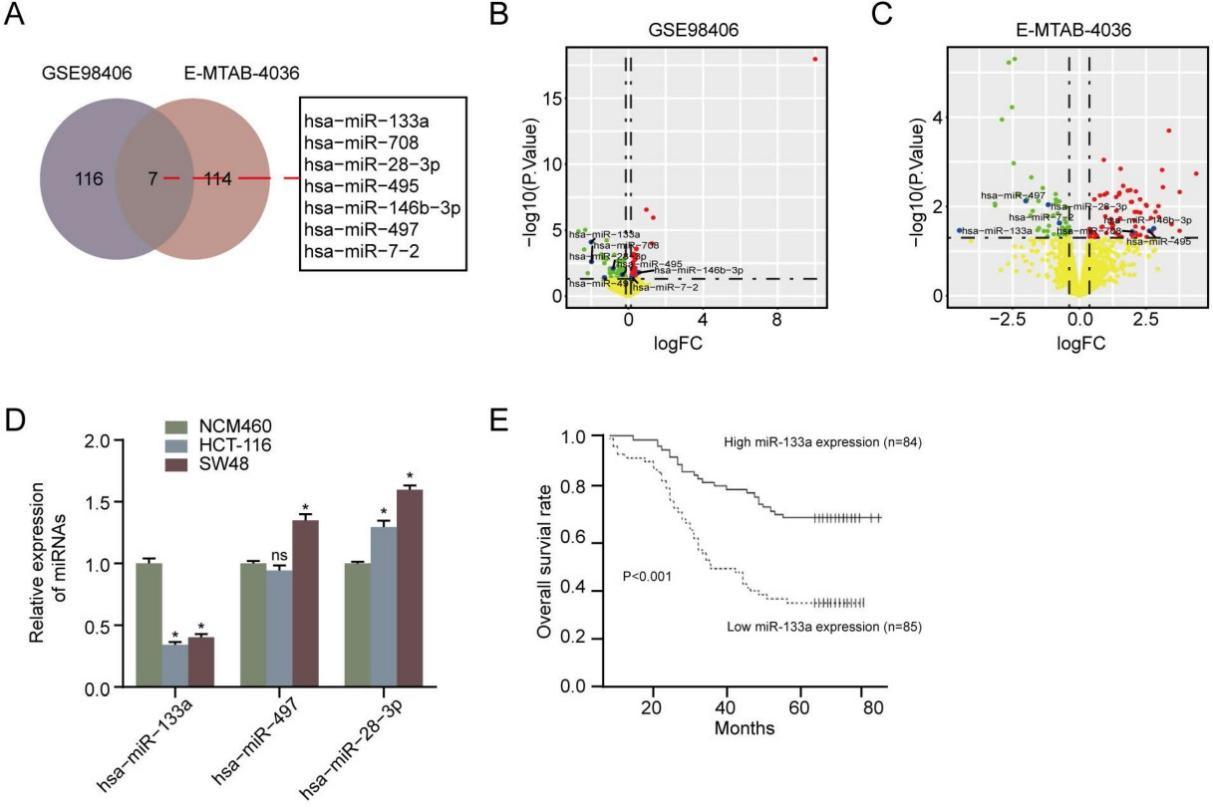
** MiR-133a expression was diminished in CRC cell lines and tissues and miR-133a low-expression was associated poor prognosis. (A)** Seven DEMs were selected in accordance to overlapping analysis of DEMs from GSE98406 and E-MTAB-4036 sets. **(B,C)** Volcano plot of Seven DEMs selected from GSE98406 and E-MTAB-4036 sets.** (D)** qRT-PCR assay of the three DEMs was performed in the two CRC cells and NCM460 cell. **(E)** Log-rank test for survival analysis according to miR-133a expression level.

We first performed overlapped miRNAs analysis between ArrayExpress database (E-MTAB-4036) and the GEO database (GSE98406) and found that seven miRNAs were DEMs in CRC tissues compared with non-CRC tissues **Figure 1A**. In addition, the clinical information of CRC patients in GEO and ArrayExpress was exhibited in **Table S1.** As a result of **Figure 1B & 1C**, we exhibited the volcano plot of seven DEMs in GSE98406 and E-MTAB-4036 sets, respectively. Moreover, RT-qPCR assay showed that miR-133a level was lower in CRC cells (HCT116 and SW48) than that in NCM460 cells (P < 0.05) (**Figure 1D**). Otherwise, we also analyzed the impact of miR-133a expression on CRC patients' prognosis in accordance to Kaplan-Meier analysis. downregulation of miR-133a was uncovered to be correlated with a shorter OS (**Figure 1E**) 20. These obtained data suggested that miR-133a played a crucial role in the tumorigenesis of CRC.

### MiR-133a hinders proliferation, invasion, migration and facilitates the apoptosis of CRC cells

**Figure 2:**
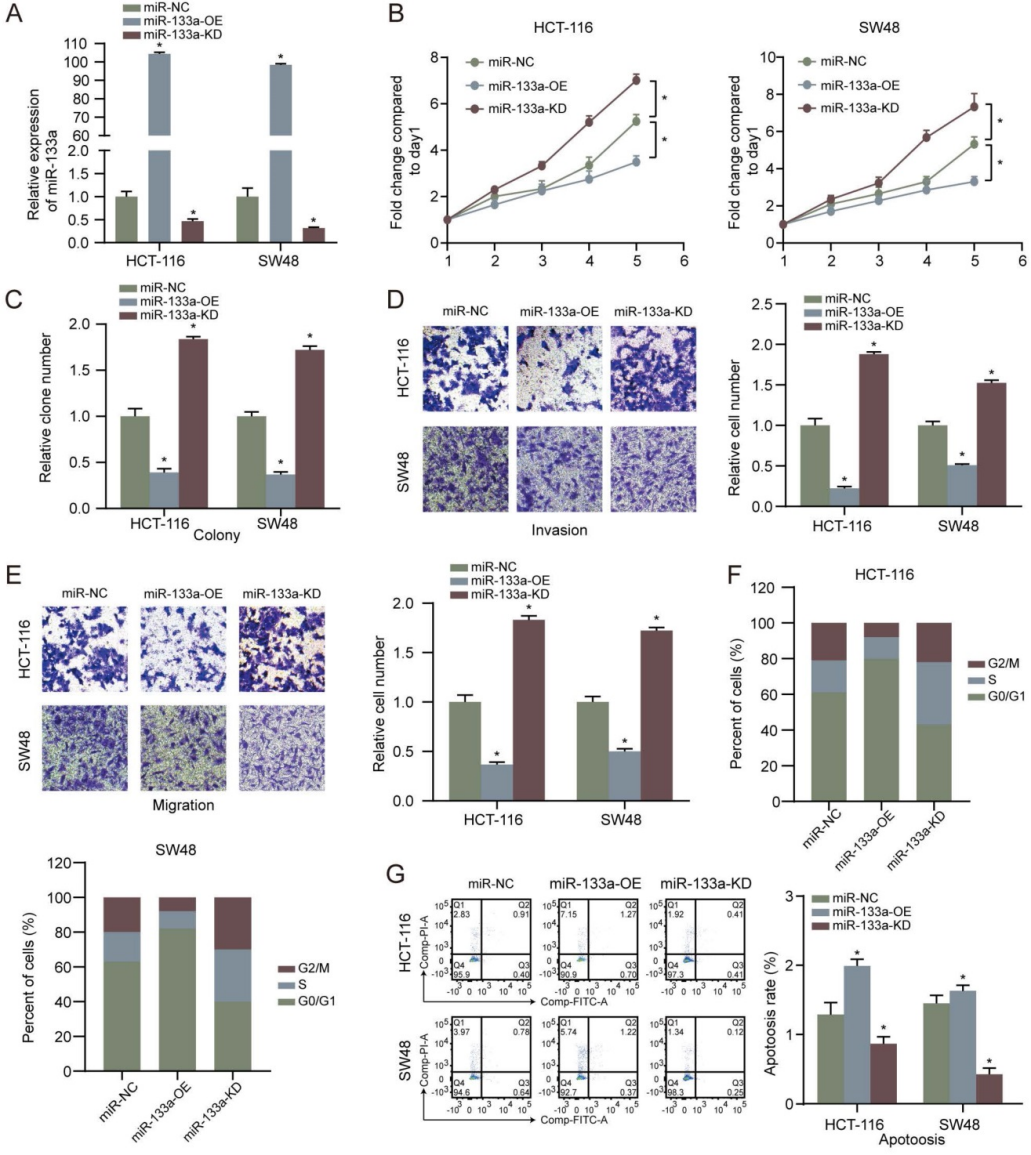
** High miR-133a expression suppresses proliferation, invasion, migration, cell cycle and augments the apoptosis of CRC cells. (A)** qRT-PCR assay for miR-133a expression level under miR-133a high-expression or reduced miR-133a expression in HCT116 and SW48 cells. **(B, C)** Cell proliferative capability was measured based on cell viability and colony formation with miR-133a high-expression or miR-133a low-expression in the both CRC cells. **(D, E)** Transwell assay was executed to analyze cell invasive and migratory ability with high miR-133a expression or diminished miR-133a expression in HCT116 and SW48 cells. **(F, G)** The impact of miR-133a low-expression or overexpression on cell cycle arrest and apoptosis in the two CRC cells was analyzed.

MiR-133a was overexpressed or low-expressed in the two CRC cells to explore the influence of miR-133a on the CRC cell proliferation, invasion, migration and apoptosis. The results revealed that miR-133a level was elevated in CRC cells with miR-133a mimics, whereas miR-133a level was reduced in CRC cells with miR-133a inhibitor (**Figure 2A**), indicating that ectopic expression of miR-133a was successfully performed. Our data revealed that overexpression of miR-133a prominently blocked the proliferation, invasion as well as migration of the two CRC cells. Whereas, downregulation of miR-133a significantly promoted the proliferation, invasion and migration of the both CRC cells (**Figure 2B-E**), implying that miR-133a may serve as a cancer suppressor for CRC. On the other hand**,** miR-133a overexpression prominently blocked cell cycle progression and reinforced CRC cells apoptosis while miR-133a downregulation clearly promoted cell cycle progression and hindered CRC cells apoptosis (**Figure 2F,G**).

### UBA2 is a direct target of miR-133a in CRC cells and restoration of UBA2 rescues the cancer inhibitory role of miR-133a in CRC cells

**Figure 3:**
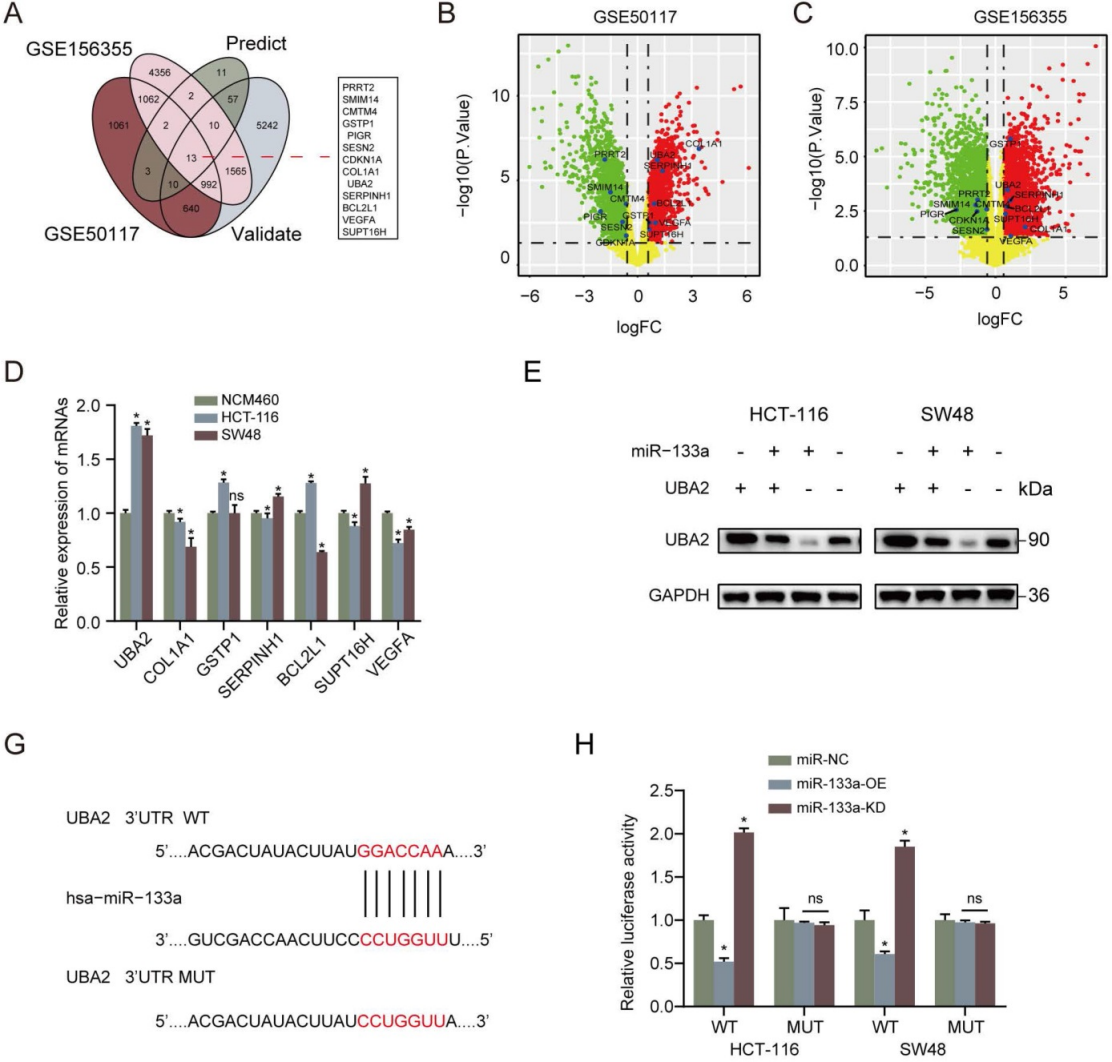
** UBA2 is a direct target of miR-133a in CRC cells. (A)** 13 common possible target genes were unveiled across DEGs' overlapping analysis from GSE50117 and GSE156355 with predicted and validated target genes. **(B, C)** Volcano plot of the unveiled 13 DEGs in GSE50117 and GSE156355, respectively. **(D)** QRT-PCR analysis for UBA2, COL1A1, GSTP1, SERPINH1, BCL2L1, SUPT16H and VEGFA in HCT116, SW48 and NCM460 cells. **(E)** Western blot assay for UBA2 with elevated miR-133a expression or reduced miR-133a expression. **(F)** MiR-133a binding site of UBA2's 3'-UTR.** (G)** pMIR-REPORT luciferase vector consisting of UBA2 3'UTR or a mutated type was co-transfected in the two CRC cells with miR-133a overexpression or miR-133a low-expression or miR-NC. Firefly luciferase activity was measured by applying Renilla luciferase activity.

**Figure 4:**
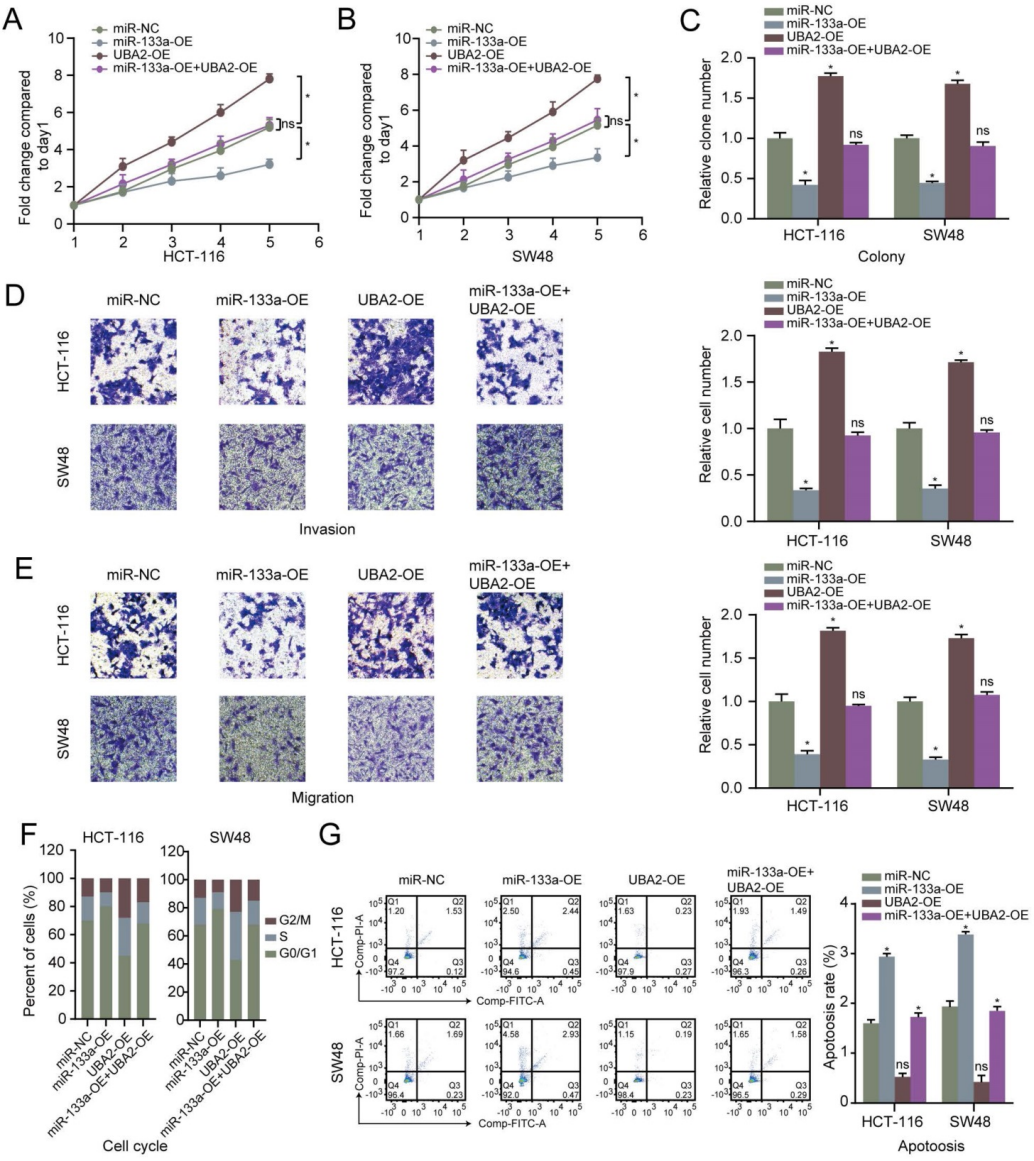
** Restoration of UBA2 rescues the cancer inhibitory role of miR-133a in CRC cells. (A-C)** The effect of miR-133a and UBA2 on CRC cell proliferative capability was analyzed according to cell viability and colony formation. **(D, E)** The effect of miR-133a and UBA2 on CRC cell invasion and migration capacity was assessed across transwell assay. **(F, G)** The effect of miR-133a and UBA2 on cycle arrest and apoptosis in CRC cell, respectively.

Furthermore, we analyzed the latent mechanisms of miR-133a' inhibitory role in CRC. 13 common possible target genes were found out according to DEGs' overlapping analysis from GSE50117 and GSE156355 with predicted and validated target genes through miRWalk database (**Figure 3A**). As for the screened 13 common target genes, seven target genes expression in CRC tissues was revealed to be higher than that in control group (**Figure 3B,C**). In addition, in terms of the seven high expression target genes, UBA2 was the most prominently upregulated in the CRC cells in comparison to NCM460 cells (**Figure 3D**). To uncover the target of miR-133a in CRC cells, firstly we determined the possible target genes of miR-133a by applying miRWalk database for miRNA target prediction. As a result, UBA2 were selected for target analysis and the underlying binding site between miR-133a and UBA2 was predicted via miRWalk database (**Figure 3F**). To further explore whether UBA2 was a direct target of miR-133a, luciferase assay was conducted. These achieved data suggested that the luciferase activity of the UBA2-WT-3'-UTR plasmid in the CRC cell line was clearly decreased by miR-133a upregulation, and was obviously raised by miR-133a downregulation, however, the luciferase activity of UBA2-MUT-3'-UTR did not change (**Figure 3G**), indicative of the direct correlation between miR-133a and the 3'UTR of UBA2 mRNA. In addition, the obtain data also indicated that decreased miR-133a expression augmented the proliferation, invasion and migration of the two CRC cells (P<0.05) (**Figure 4A-E**). The data also exhibited that miR-133a high-expression obviously attenuated cell cycle and enhanced CRC cells' apoptosis, which was prominently rescued by UBA2 high-expression (**Figure 4F,G**). Afterwards, we detected the relative expression levels of UBA2 with miR-133a high-expression or miR-133a low-expression in accordance to western blot analysis. These results exhibited that miR-133a upregulation evidently raised UBA2 expression and, while miR-133a low-expression had a reverse outcome (**Figure 3E**). Meanwhile, low UBA2 expression was correlated with good overall survival rate in CRC patients 21 (**Figure S1A**) and UBA2 was negatively associated with miR-133a albeit there is not significant in starbase database (**Figure S1B**). Based on these findings, we concluded that miR-133a might exert its role in CRC cells across directly regulating UBA2.

### MiR-133a is a direct target of FOXD3

**Figure 5:**
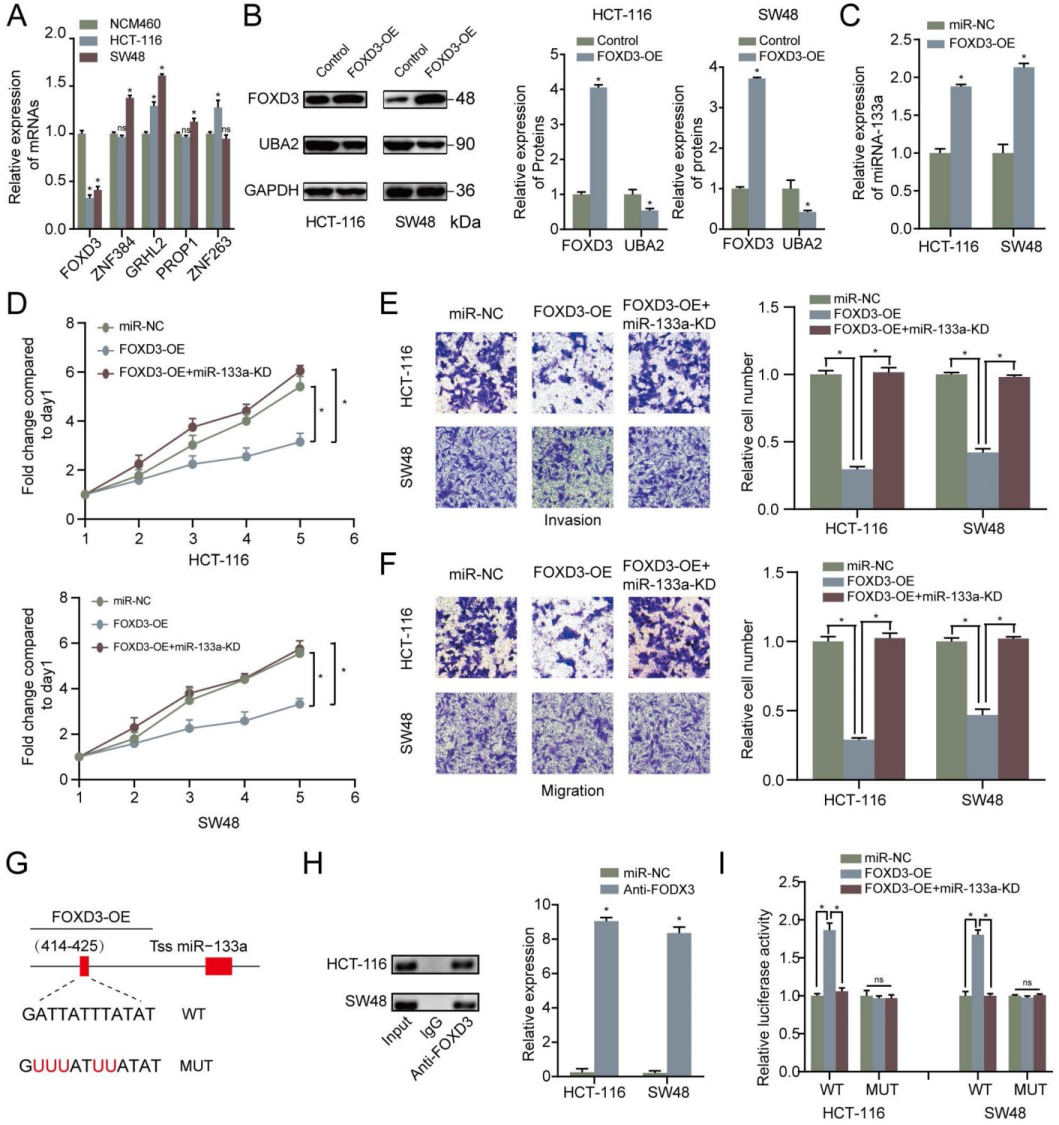
** MiR-133a is a direct target of FOXD3. (A)** qRT-PCR assay for FOXD3, ZNF384, GRHL2, PROP1 and ZNF263 expression level. **(B)** The effect of FOXD3 high-expression on miR-133a in the two CRC cells was measured via western blot assay. **(C)** MiR-133a was obviously raised under FOXD3 high-expression. **(D)** High FOXD3 expression significantly augmented the number of CRC-derived cell colonies formed, which was indicated by colony formation assay, and the effect was obviously reversed by miR-133a low-regulation. **(E, F)** Increased FOXD3 expression obviously heightened cell invasive and migratory capacity of HCT116 and SW48 cells, which was clearly rescued by diminished miR-133a expression based on transwell assay. **(G)** A schematic exhibited the proximal region of the miR-133a promoter and the region was detected by ChIP assay. **(H)** ChIP was adopted to validate the binding correlation between FOXD3 and the miR-133a promoter in the two CRC cells. **(I)** Luciferase activity was obviously augmented in the WT miR-133a promoter under high FOXD3 expression and the effect was obviously rescued by low miR-133a expression. Whereas, no obvious difference was found under mutation of FOXD3 binding site at 414-425 bp. *P<0.05, **P<0.01.

To find out the upstream modulatory molecules of miR-133a, FOXD3 as a TF targeting promoters of miR-133a was identified by using JASPAR. Firstly, qRT-PCR assay was adopted to analyze the expression level of the top five potential TFs that could regulate the expression of miR-133a. We discovered that the expression level of FOXD3 was the most significantly high-expressed molecule in the two CRC cells compared with that in NCM460 cells (**Figure 5A**). Western blot analysis was adopted to measure the relative expression of FOXD3 and UBA2 with FOXD3 high-expression. The western blot analysis result manifested that UBA2 expression levels were decreased with FOXD3 upregulation in the two CRC cells (**Figure 5B**). As demonstrated in **Figure 5C**, QRT-PCR analysis proved that FOXD3 high-expression obviously increased the miR-133a expression in the two CRC cells. The evidence also revealed that high-expression of FOXD3 could suppress proliferation, invasion and migration of CRC cells, and this effect could be reversed by miR-133a downregulation (P<0.05) (**Figure 5D-F**).

**Figure 6:**
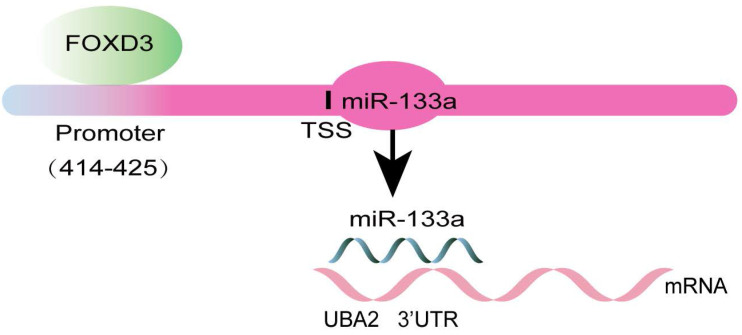
Schematic diagram of the present study.

Otherwise, ChIP assay was executed to verify whether FOXD3 transcriptionally regulated miR-133a expression. Binding sites of FOXD3 inside the miR-133a promoter region was predicted according to JASPAR database. The ChIP assay identified the region at 414-425bp upstream of the pre-miR-133a promoter region as a target of FOXD3 (**Figure 4F, G**). Furthermore, luciferase activity in the WT miR-133a promoter was apparently increased under upregulation of FOXD3, and the effect was clearly rescued by miR-133a mimic. Whereas, no obvious difference was found under mutation of FOXD3 binding site at 414-425bp (**Figure 5G**). In addition, high FOXD3 expression was related with good overall survival rate (**Figure S1C**) and the expression of FOXD3 was positively correlatively with miR-133a (**Figure S1D**) in starbase database. Those results indicated that FOXD3-induced miR-133a block inhibits malignant progression of colorectal cancer through regulating UBA2 (**Figure 6**).

## Discussion

Plenty of studies demonstrated that miRNAs were strongly correlated with tumorigenesis 22-24. Otherwise, evidence suggested that miRNAs correlated with treatment of different types of tumors. For instance, Petrovic et al. revealed miRNAs as potential treatment targets and treatment options in cancer 25. Jiang et al. found the clinical value of circular RNAs and autophagy-related miRNAs in the diagnosis and treatment of pancreatic cancer 26. On the other hand, we found that miR-133a was remarkably reduced in CRC samples via a comprehensive bioinformatics approach, but the regulatory mechanism of the miR-133a in CRC keep still unclear.

In the present study, we investigate the function and mechanism of miR-133a in CRC. Consistent with our findings, miR-133a low-expression was also reported in gastric cancer 10, prostate cancer 27, esophageal cancer 28, and cervical cancer 29. These results suggested that upregulation of miR-133a may serve as a repressor in various carcinomas. In the present paper, miR-133a high-expression was also verified to hinder the proliferation, invasion, migration and promote apoptosis of CRC cells (**Figure 2**). In this study, the data of **Figure 1E** exhibited that downregulation of miR-133a was tightly related to a shorter OS (P < 0.05), suggesting that miR-133a may act as an effective biobiomarker for predicting CRC patients' prognosis. On the other hand, a lot of studies suggested that UBA2 functioned as an oncogenic gene. For example, Cheng et al. suggested that knockdown of UBA2 inhibited CRC cell invasion and migration by downregulation of the Wnt/beta-catenin signaling pathway 30. Li et al. indicated that UBA2 promoted cell migration and invasion through Wnt/β-catenin signaling in gastric cancer 31, which were consistent with the findings in the present study. Additionally, the result of **Figure 4** showed that UBA2 could promote the proliferation, invasion, migration and block apoptosis of CRC cells.

Numerous studies revealed that TFs were closely involved in different cancers. For example, Liu et al. found that TF c-Maf was a checkpoint that programs macrophages in lung cancer 32. Jiang et al. indicated that TF NFAT5 promoted pancreatic cancer progression through transcription of PGK1 33. Seoane et al. suggested that POU1F1 TF promoted breast cancer metastasis by recruitment and polarization of macrophages 34. Liu et al. revealed expression and clinical significance of TF 4 in epithelial ovarian cancer 35. In this study, we first predicted TFs that targeted the promoter of miR-133a via JASPAR database. Following that, we analyzed the influence of high FOXD3 expression on miR-133a expression and miR-133a expression by qRT-PCR or western blot assay. Furthermore, the impact of increased FOXD3 expression on proliferation, invasion and migration ability of CRC cells was also analyzed. Based on the result of Figure** 5D-F,** high FOXD3 expression could suppress proliferation, invasion and migration of CRC cells.

## Conclusion

In conclusion, these data manifested that FOXD3/miR-133a/UBA2 axis played a key role in the growth and progression of CRC. Our study suggested that FOXD3/miR-133a/UBA2 axis may have a great potential as a therapeutic target for CRC patients. The inhibitor of UBA2, and activator of FOXD3 and miR-133a may have a protective impact on CRC patients, which deserves further exploration and development.

## Supplementary Material

Supplementary figure 1.

Supplementary table 1. The clinical information of CRC patients in GEO and ArrayExpress.

## Figures and Tables

**Table 1:** All special primers were used in this study

Primer	Sequence
MiR-133a	Forward: 5′-ATAAGAATGCGGCCGCATTCCAAACTAGCAGCACTA-3′
	Reverse: 5'-AGCTTTGTTTAAACTTAACCATTCTAGCTTTTCC-3'
U6	Forward: Universal U6 Primer F (human)
	Reverse: Universal PCR Primer R
GAPDH	Forward: 5′ GCATCTTCTTGTGCAGTGCC, 3′
	Reverse: 5' TACGGCCAAATCCGTTCACA, 3'
MiR-497	Forward: 5′-GTGCAGGGTCCGAGGT-3′
	Reverse: 5′-TAGCCTGCAGCACACTGTGGT-3′
MiR-28-3p	Forward: 5′-CGCGCACTAGATTGTGAGCT-3′
	Reverse: 5′-AGTGCAGGGTCCGAGGTATT-3′
UBA2	Forward: 5′-CACAGGTTGCCAAGGAA-3′
	Reverse: 5′-GACACTCATAACACTCGGTCA-3′
COL1A1	Forward: 5′-CTGCTGGACGTCCTGGTGAA-3′
	Reverse: 5′-ACGCTGTCCAGCAATACCTTGAG-3′
GSTP1	Forward: 5′-CCCTACACCGTGGTCTATTTCC-3′
	Reverse: 5′-CAGGAGGCTTTGAGTGAGC-3′
SERPINH1	Forward: 5'-CAGAAGTTTCTCGGGACGGG-3'
	Reverse: 5'-GCCTGCCTTTTTCATTCTGGG-3'
BCL2L1	Forward: 5′-GCAGTTCAAACTCGTCGCCT-3′'
	Reverse: 5′-TCGAAGAACTGTTCCAGCCA-3′
SUPT16H	Forward: 5′-CGG GCA GCA TTA CTT ACA GA-3′
	Reverse: 5′-TTC AGT CAA TCG CCT CTT TG-3′
VEGFA	Forward: 5ʹ-TGCCATCCAATCGAGACCCTG-3ʹ
	Reverse: 5ʹ-GGTGATGTTGGACTCCTCAGTG-3ʹ
FOXD3	Forward: 5′GGTAGCGTTAGCGATATGTTC 3′
	Reverse: 5′ACGTCGCTATCCTTCTCTTC 3′
ZNF384	Forward: 5′-GTCTCAGGTCAGATCGAGAACA-3′
	Reverse: 5′-ACTCTGTGTCCATACTGATGCC-3′
GRHL2	Forward: 5'-GGAAATCTAG CCCTGGGTTT G-3'
	Reverse: 5'-TCAGGGAGGA ACGCACTGA-3'
PROP1	Forward: 5'-AGCAACGGAAGCAAGAGCG-3'
	Reverse: 5'-GGGTGAGGGAAGCAGGTCA-3'
ZNF263	Forward: 5'-AGGATGGGATTCTGAGGTA-3'
	Reverse: 5'-ACAAGGGACATAAATGGAGT-3'
