# Supplementary material for: FOXD3-induced miR-133a blocks progression and metastasis of colorectal cancer through regulating UBA2
Source: J Cancer. 2021 Aug 25;12(20):6145–54. doi: 10.7150/jca.60647 (PMC8425194; doi:10.7150/jca.60647)

Figure S1 **A**, the relationship between UBA2 and CRC patients' prognosis. **B**, the correlation between UBA2 and miR-133a was analyzed. **C**, the linkage between FOXD3 and CRC patients' prognosis. **D**, the correlation between FOXD3 and miR-133a was analyzed.

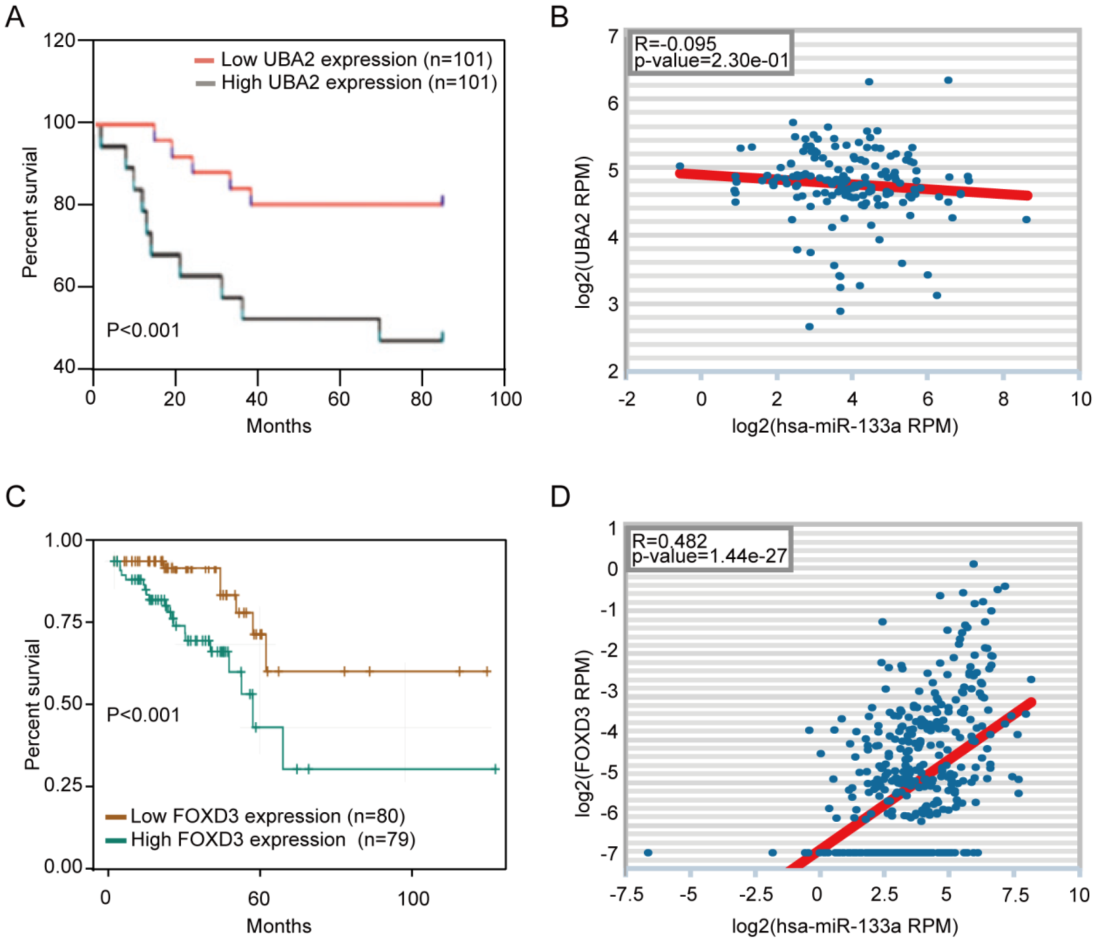

Supplement: Supplementary file 1 — Supplementary figure 1. [file jcav12p6145s1.pdf]
